# Analysis of redox status and HDL subclasses in patients with lymphoma and the associations with FDG-PET/CT findings

**DOI:** 10.3389/fonc.2023.1221414

**Published:** 2023-10-30

**Authors:** Bosa Mirjanic-Azaric, Sinisa Stankovic, Lana Nezic, Zana Radic Savic, Dragana Malcic- Zanic, Ranko Skrbic, Jelena Kotur-Stevuljevic, Natasa Bogavac-Stanojevic

**Affiliations:** ^1^ Department of Medical Biochemistry, Faculty of Medicine, University of Banja Luka, Banja Luka, Bosnia and Herzegovina; ^2^ Institute of Laboratory Diagnostic, University Clinical Centre of the Republic of Srpska, Banja Luka, Bosnia and Herzegovina; ^3^ Institute of Nuclear Medicine, University Clinical Centre of the Republic of Srpska, Banja Luka, Bosnia and Herzegovina; ^4^ Faculty of Medicine, University of Banja Luka, Banja Luka, Bosnia and Herzegovina; ^5^ Department of Pharmacology, Toxicology and Clinical Pharmacology, Faculty of Medicine, University of Banja Luka, Banja Luka, Bosnia and Herzegovina; ^6^ Department of Pediatrics, Faculty of Medicine, University of Banja Luka, Banja Luka, Bosnia and Herzegovina; ^7^ Academy of Sciences and Arts of the Republic of Srpska, Banja Luka, Bosnia and Herzegovina; ^8^ Department of Medical Biochemistry, University of Belgrade-Faculty of Pharmacy, Belgrade, Serbia

**Keywords:** redox status, HDL subclasses, SUVmax, Hodgkin lymphoma, non-Hodgkin lymphoma

## Abstract

Newer research points to alterations in the plasma redox status and the HDL subclass distributions in cancer. We aimed to assess the redox status and the HDL subclass distributions, lipids, and inflammatory markers in lymphoma patients in order to determine whether they were correlated with changes in FDG-PET/CT scans. At the beginning of this study, redox status, HDL subclasses, lipids, and inflammation biomarkers were determined in 58 patients with lymphoma (Hodgkin lymphoma, n=11 and non-Hodgkin lymphoma, n=47), and these same measurements were reassessed during their ensuing treatment (in 25 patients). Initially, the total oxidation status (TOS), the prooxidant–antioxidant balance (PAB), the OS index (OSI), the total protein sulfhydryl groups (SH-groups), and the advanced oxidation protein products (AOPP) were significantly higher in lymphoma patients as compared to healthy subjects, but the total antioxidant status (TAS) was significantly reduced. The PAB had a strong correlation with the CRP and interleukin-6 (rho=0.726, p<0.001; rho=0.386, p=0.003). The correlations between these parameters and the maximum standardized uptake values (SUVmax) were: PAB, rho=0.335 and p=0.010; SH-groups, rho=0.265 and p=0.044; CRP, rho=0.391 and p=0.002; HDL3b, rho=0.283 and p=0.031; HDL2b, rho= -0.294 and p=0.025; and HDL size, rho= -0.295 and p=0.024. The reductions in SUVmax between two follow-up points were associated with increases in the OSI, TOS, and SH-groups, as well as a reduction in the PAB and TAS. In conclusion, the redox parameters in patients with lymphoma were consistent with FDG-PET/CT findings. Targeting the redox status parameters and the HDL subclasses could be potential strategies in the molecular fight against lymphoma.

## 1 Introduction

Lymphomas represent a very heterogeneous group of hematological malignancies that are classified as non-Hodgkin lymphoma (NHL), which accounts for approximately 90% of cases, and Hodgkin lymphoma (HL), which has a significantly lower incidence rate at approximately 10% (1). Though many risk factors for these diseases have been identified (e.g., environmental, infectious, inflammation, immunodeficiency, and genetic), the exact etiology of lymphoma remains ambiguous. Oxidative stress (OS) has been found to play an important role in carcinogenesis (2). The long-term effect of OS leads to the damage and dysfunction of vital cellular structures and macromolecules, such as proteins, DNA, and lipids, which ultimately contribute to the pathogenesis of numerous serious diseases, including cancer (2). There is accumulating evidence that reactive oxygen species (ROS) act as either tumor-promoting or tumor-suppressing agents, with abundant evidence supporting both arguments (3). Nonetheless, increased levels of ROS have been found to modify the regulation of the signaling pathways involved in cell proliferation, growth, survival, and apoptosis (2, 4). However, the definitive processes by which ROS cause cell proliferation and cell death remain undefined.

Extensive research has been focused on the anti-oxidative and anti-inflammatory roles of high-density lipoprotein (HDL). The antioxidant capacity of HDL depends on the presence of antioxidant enzymes, primarily on the paraoxonase 1 activity and the distribution of the HDL particles (5–7). Moreover, paraoxonase 1 is involved in the inactivation of prooxidant and pro-inflammatory mediators, the regulation of cell proliferation, and scavenges lipid-soluble carcinogenic radicals (6). HDL particles may be different densities, sizes, and compositions, which influences the differences in their vasoprotective functions. Namely, HDL3 has a greater antioxidant activity than HDL2, and HDL3 has a higher paraoxonase 1 activity (8, 9). Cholesterol is closely related to malignant cell survival, and HDL mediated cholesterol transport may have a significant role in tumor development (10). Studies have shown that HDL particles stimulated the growth of breast cancer cells *in vitro* and increased the aggressiveness of malignant tumors in mice (11, 12). Additionally, it was reported that breast cancer cells acquired cholesterol, primarily from HDL3 particles, to proliferate. It was postulated that the uptake mechanism was mediated by CD36 and LIMPII analogous-1 (CLA-1), a human homolog of the rodent receptor SR-B1 receptor (13).

Numerous studies have confirmed the linkage between OS, inflammation, and cancer. Namely, OS could lead to chronic inflammation, which could then mediate cancer development and extensive research has demonstrated an association with interleukin-6, C-reactive protein (CRP), and ferritin, with cancer (14).

In addition, magnesium deficiency upregulates proinflammatory cytokines and increases OS, which could contribute to the development of NHL (15, 16).

To better understand the natural course of lymphoma and promote better clinical decisions, it is important to observe the correlation between redox biomarkers, HDL subclasses with inflammatory parameters, and their relation to the progression of lymphoma changes, which can be monitored by positron emission tomography/computed tomography (PET/CT).

The aim of the present study was to analyze biomarkers of redox status and the HDL subclasses, as well as the inflammatory parameters, in patients with previously untreated lymphoma prior to and during their ensuing treatment. Also, we analyzed whether these circulatory markers associated with OS and inflammation could be associated with 18 F-2-fluoro-2-deoxy-D-glucose (FDG) PET/CT findings.

## 2 Materials and methods

### 2.1 Patients

Fifty-eight newly diagnosed lymphoma patients over the age of 18 were consecutively enrolled in the study, which was conducted at the University Clinical Centre of the Republic of Srpska, Banja Luka (Bosnia and Herzegovina) from July 2020 to April 2022. The study was conducted in accordance with the Declaration of Helsinki and approved by the Ethics Committee of the University Clinical Centre of the Republic of Srpska, Banja Luka (No 01-19-51-2/20), and at the Faculty of Medicine at the University of Banja Luka (No 18/4.3.95/2020). Informed consent was obtained from all subjects involved in the study. All patients enrolled in the study met the inclusion criteria, were over the age of 18, had no previous history of malignancy, moreover, lymphoma was a primary malignancy, and no current infections; and their diagnosis had been confirmed by a tissue biopsy following a histopathological diagnosis *de novo*, immunohistochemistry, and flow cytometry, according to WHO’s expert guidelines (17). Of the 58 patients, 11 patients had HL, and 47 had NHL. The stage of the disease was determined using the Lugano classification system (18). According to its patohystologic features, the clinical course of the disease, as well as, the way of growth and spread, and in agreement with literature data the NHL were divided into two groups: aggressive lymphomas with rapid growth and fast spread, and indolent slow-growing lymphomas with slow clinical onset (19).

The control group consisted of 58 matching volunteers who met the following inclusion criteria: within ( ± 10 years of age); both sexes; no history of malignancy; no history of chronic diseases or acute infection in the last 3 months prior to enrollment in the study; clinical laboratory parameters within the reference range; and non-pregnant and non-lactating. Patients were treated according to the international guidelines for lymphoma, which was accepted at a national level based on drug availability. All patients with NHL were treated with the first-line with immuno-chemotherapies: the R-CHOP regimen (*i.e.*, rituximab, cyclophosphamide, doxorubicin, vincristine, prednisone) or an equivalent regime, such as R-EPOCH (i.e., rituximab, etoposide, doxorubicin, vincristine, prednisone cyclophosphamide) (20). First-line treatments for HL included the ABVD protocol (i.e., doxorubicin, bleomycin, vinblastine and dacarbazine), or in advanced stages, BEACOPP (*i.e*., bleomycin, etoposide, doxorubicin, cyclophosphamide, vincristine, procarbazine and prednisone) (21, 22).

Immuno-chemotherapy started after a baseline FDG-PET/CT lymphoma staging. As per local treatment protocols, the course of the disease as well as the treatment responses were assessed by FDG-PET/CT after four cycles of the treatment (point of reassessment).

According to the protocol, all the patients (58) had an FDG-PET/CT scan at the baseline but only 25 according to the protocol in the middle of the treatment therapy (point of reassessment). During the study period and up until the final assessments, 23 patients (39.6%) withdrew due the COVID-19 pandemic, and ten patients died: six from their COVID-19 infection, while four patients died during febrile neutropenia with signs of septic shock, but without a microbiologically confirmed etiological factor.

The demographic and clinical data for the participants, including age, sex, and clinical stage, were taken from medical history.

### 2.2 Laboratory analyses

The patients’ blood samples were obtained before the FDG-PET/CT, after overnight fasting (first time-point) and at the middle of the treatment immuno-chemotherapy (point of reassessment, second time-point) also while fasting, the same day of (and prior to) the FDG-PET/CT diagnostics. Immediately after venipuncture, samples were centrifuged, and the plasma and serum were separated and stored at -80°C until analysis.

The total antioxidant status (TAS), total oxidant status (TOS), advanced oxidation protein products (AOPP), paraoxonase 1, prooxidant–antioxidant balance (PAB), and total protein sulfhydryl groups (SH-groups) were measured on a ILab 300+ (Instrumentation Laboratory, Milan, Italy) and then were described and published elsewhere (23). The OS index (OSI) was obtained by the following calculation: OSI (arbitrary unit (AU))=[(TOS, mmol H_2_O_2_ Equiv./L)/(TAS, mmol Trolox Equiv./L)].

Plasma HDL particles were separated using a non-denaturing 3%–31% polyacrylamide gradient gel electrophoresis method. The HDL particle size determination and subclass analyses were determined based on the migration distance of the major peaks in the densitometric profile, from the calibration curve based on the high-molecular-weight protein standards. A relative proportion HDL subclasses were determined by analyzing areas under the peaks that corresponded to the known ranges of the particle diameters: HDL 2b (9.7–12.0 nm), HDL 2a (8.8–9.7 nm), HDL 3a (8.2–8.8 nm), HDL 3b (7.8–8.2 nm), and HDL 3c (7.2–7.8 nm) (24).

Interleukin-6 and ferritin were analyzed by ADVIA Centaur XP (Siemens Healthineers USA, United States). Total cholesterol, triglycerides, low-density lipoprotein cholesterol (LDL-C), high-density lipoprotein cholesterol (HDL-C), CRP, albumin, and magnesium were determined by routine methods on an Alinity Abbott analyser. The redox status and HDL subclasses were measured at the University of Belgrade’s Faculty of Pharmacy, and routine biochemical tests were performed in the Department of Laboratory Diagnostics at the University Clinical Center of the Republic of Srpska, Banja Luka.

### 2.3 FDG -PET/CT scan

A baseline and a mid-point FDG-PET/CT (i.e., during treatment but after immuno-chemotherapy) were performed on the same scanner (Discovery 610, GE Healthcare, Milwaukee, WI, USA). After overnight fasting and with an optimal blood glucose level lower than 7.5 mmol/L, the patients were injected intravenously with 3.5 MBq/kg of 18 F-FDG (18 F-2-fluoro-2-deoxy-D-glucose). Images were acquired from the skull base to the upper thighs, 60 minutes after the injection. Images were then corrected for attenuation with low-dose CT data, reconstructed with a three-dimensional iterative algorithm, and, finally, fused with CT images. Maximum standardized uptake value (SUV max) as a marker of tumor glucose metabolism was measured on the dominant lesion or the hot-test focus of uptake, SUVmax = maximum activity in region of interest (ROI) (kBq)/injected dose (MBq) × body weight (kg) (25). Assessment of the therapeutic response was conducted using the Deauville score five-point scale (Deauville criteria) (26). According to the Lugano Classification, a score of 1 or 2 signifies a complete metabolic response, a score of 3 or 4 indicates a partial metabolic response, and a score of 5 denotes progressive metabolic disease. The patients were categorized into three groups: complete metabolic responders, partial metabolic responders, and non-responders/progressive metabolic disease. The FDG-PET/CT scan was conducted in the Department of Nuclear Medicine at the University Clinical Center of the Republic of Srpska, Banja Luka.

### 2.4 Statistical analysis

The data distribution was tested using the Kolmogorov–Smirnov test. Depending on the type of variables and the normality of the distribution, Student’s t-test for independent data and the Mann–Whitney U test were used to analyze the differences in the data between patients and controls. Categorical variables were tested by the Chi-squared test. The correlations between the variables were estimated using Spearman’s correlation coefficient (Rho). The Wilcoxon signed-rank test and the Student’s t-test for dependent data were used to analyze changes between the two repeated observations in the patient group. Moreover, to analyze whether the changes in the examined parameters between the two time-points were interrelated, univariate, or multivariate linear, the fixed-effects regression model for panel data was used. The data were shown as mean ± standard deviation for normally distributed variables. The median for independent data and the median of difference for dependent data with an interquartile range were presented for non-normally distributed variables. Relative or absolute frequencies were shown for categorical variables. Additionally, analysis of covariance (ANCOVA) and Quade’s test were carried out to investigate the influence of age as confounder on the difference in normally distributed and skewed variables, respectively.

The statistical analyses were performed with PASW Statistics, v. 27, software (Chicago, Illinois, USA). A two-tailed p-value ≤0.05 was considered statistically significant.

## 3 Results

The study population consisted of 58 patients with lymphoma versus healthy subjects (**Table 1**). The patients with lymphoma were significantly older, as compared to the healthy subjects but sex balanced. The assessment of the OS parameters at the first time-point (*i.e*., baseline value, before treatment) showed that the TAS values were significantly reduced, in contrast to the parameters of the TOS, AOPP, PAB, OSI, and SH-groups, which had concentrations significantly higher, as compared to the control group. The paraoxonase 1 activity was lower than in the control group [219 (135–424) vs. 255 (182–531)] and, although not statistically significant, showed a trend towards reduced antioxidants in the malignant tumors.

**Table 1:** Clinical data and laboratory parameters of study population prior to treatment.

Parameters	Patients (n=58)	Control group (n=58)	*p*-value
**Male sex, n (%)**	28 (46.7)	32 (53.3)	0.457
**Age, years**	58 ± 15.9	50 ± 12.2	0.001
Medical history of patients with diagnosed lymphoma			
**HL, n (%)**	11 (19.0)	/	/
**NHL, n (%)**	47 (81)	/	/
**Follicular NHL, n**	21	/	/
**DLBCL, n**	14	/	/
**SLL, n**	4	/	/
**MCL, n**	2	/	/
**ALCL, n**	2	/	/
**LPL, n**	1	/	/
**MZL, n**	1	/	/
**PTCL, n**	1	/	/
**Burkitt NHL, n**	1	/	/
**SUVmax (g/ml) **	10.3 ± 8.33	/	/
Redox parameters			
**Paraoxonase 1 (U/L)**	219 (135-424)	255 (182-531)	0.081
**TAS (mmol/L)**	994 (808-1136)	1165 (951-1288)	0.001
**SH-groups (mmol/L)**	0.383 (0.271-0.451)	0.279 (0.235-0.319)	<0.001
**TOS (mmol/L)**	13.0 (8.8-18.6)	8.8 (5.8-14.3)	0.004
**PAB (U/L)**	132.50 (109-171)	94.05 (77-105.50)	<0.001
**OSI (AU)**	0.012 (0.008-0.022)	0.008 (0.005-0.014)	0.002
**AOPP (μM)**	52.8 (46.8-65.0)	45.8 (34.6-54.6)	<0.001
Inflammatory parameters			
**CRP (mg/L)**	4.25 (1.05-11.72)	0.80 (0.40-2.87)	<0.001
**Interleukin 6 (pg/mL)**	2.35 (1.30-5.40)	1.15 (0.65-2.45)	<0.001
**Ferritin (ng/mL)**	97 (37-262)	28 (13-64)	<0.001
Lipid parameters			
**Cholesterol (mmol/L)**	4.82 ± 1.24	5.51 ± 0.95	0.001
**HDL-C (mmol/L)**	1.16 ± 0.39	1.39 ± 0.39	0.002
**LDL-C (mmol/L)**	3.11 ± 1.05	3.56 ± 0.98	0.019
**Triglycerides (mmol/L)**	0.63 (0.44-0.53)	1.08 (0.65-1.56)	<0.001
**HDL size (nm)**	9.75 ± 0.99	9.53 ± 0.87	0.208
**HDL2b (%)**	41.7 ± 13.2	38.0 ± 9.9	0.100
**HDL2a (%)**	20.6 ± 4.4	21.7 ± 6.3	0.280
**HDL3a (%)**	13.8 ± 3.7	17.0 ± 5.4	<0.001
**HDL3b (%)**	8.9 ± 3.6	10.5 ± 3.9	0.022
**HDL3c (%)**	15.0 ± 10.9	12.6 ± 6.6	0.157
**HDL3 (%)**	37.7 ± 14.4	40.1 ± 12.1	0.331
Trace element and albumin			
**Magnesium (mmol/L)**	0.78 ± 0.08	0.83 ± 0.04	0.004
**Albumin (g/L)**	44.4 ± 4.84	47.0 ± 2.78	0.002

Categorical data are presented as absolute frequencies (percentages) and analyzed by the Chi-squared test. Normally distributed data are presented as means ± SD and compared by Stu-dent’s t-test for independent data, and continuous data with skewed distribution are presented as median and interquartile range and compared by the Mann–Whitney U test; p, level of significance. NHL, non-Hodgkin lymphoma; DLBCL, diffuse large B cell lymphoma; SSL, small lymphocytic lymphoma; LPL, lymphoplasmacytic lymphoma; MCL, mantle cell lymphoma; MZL, marginal zone lymphoma; PTCL, peripheral T-cell lymphoma; ALCL, anaplastic large-cell lymphoma; paraoxonase 1 activity; TAS, total antioxidant status; TOS, total oxidation status; AOPP, advanced oxidation protein products; PAB, prooxidant–antioxidant balance; SH-groups, total protein sulfhydryl groups; OSI, oxidative stress index; CRP- C-reactive protein,LDL-C, low-density lipoprotein-cholesterol; HDL-C, high-density lipoprotein-cholesterol; HDL high-density lipoprotein; SUVmax, maximum standardized uptake value.

Patients had lower lipid levels than the controls but expectedly higher inflammatory parameters (**Table 1**). The analysis of the HDL lipoprotein subclass profile showed that the patients had significantly smaller proportions of the HDL3a and HDL3b particles, than the controls. The difference in normally distributed parameters (lipid and proportions of HDL subclasses) between healthy and lymphoma subjects was evaluated by ANCOVA, with age included as covariates. The adjusted means are shown in **Supplementary Table 1**. The parameter difference between healthy and lymphoma subjects was not confounded by age. Additionally, Quade’s test was applied to compare redox and inflammatory parameters and triglycerides between two groups with age as confounder (**Supplementary Table 2**). As in the previous case, age did not influence a difference in redox and inflammatory parameters (**Supplementary Table 2**).

**Figure 1:**
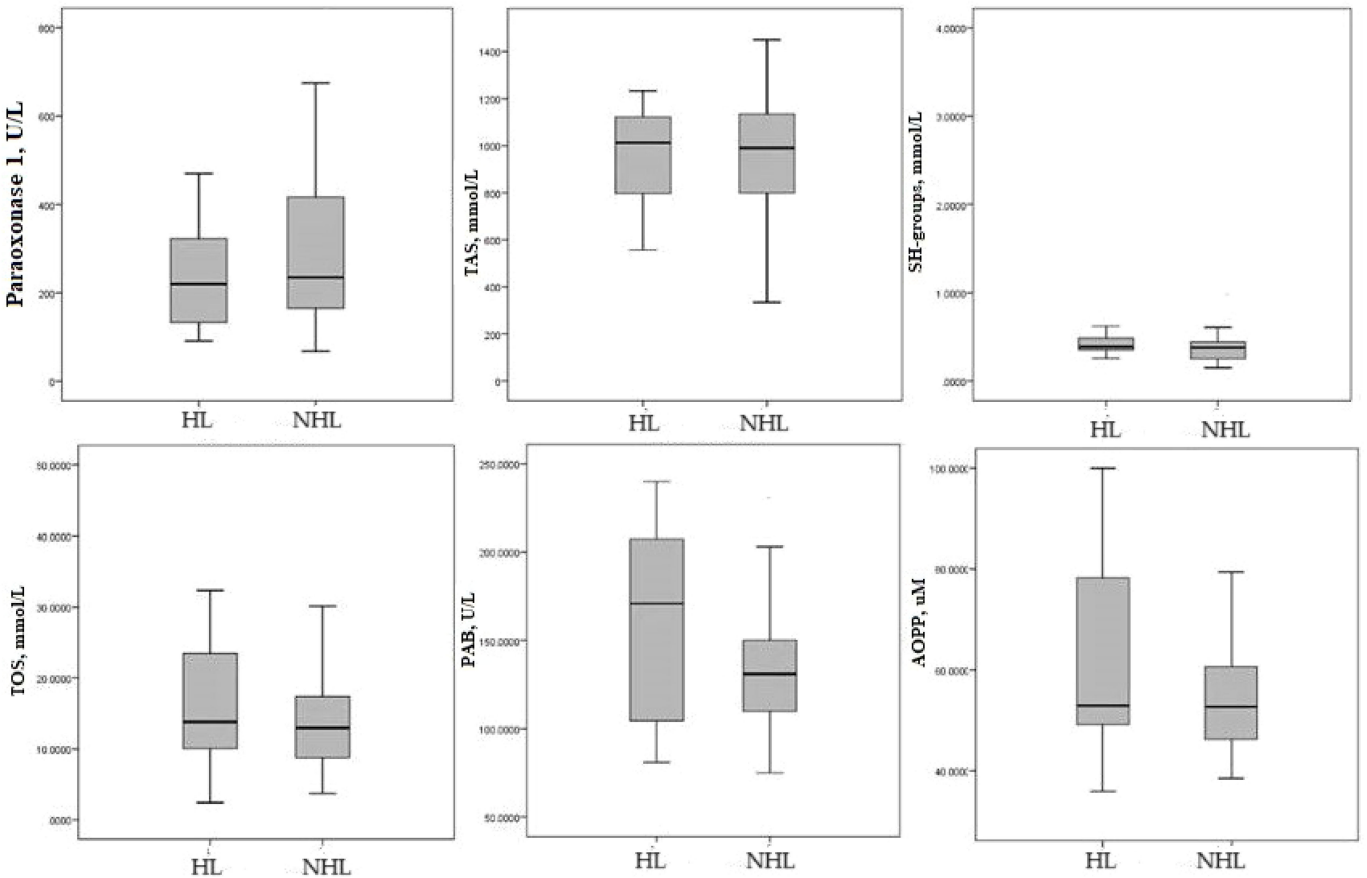
Median of redox parameters Hodgkin lymphoma and non-Hodgkin lymphoma. HL-Hodgkin lymphoma; NHL non-Hodgkin lymphoma; TAS, total antioxidant status; SH-groups, total protein sulfhydryl groups; TOS, total oxidation status; PAB, prooxidant–antioxidant balance; AOPP, advanced oxidation protein products.

Additional, **Figure 1** shown that there was no difference in the values of redox parameters between HL and NHL.

Also, significantly higher values of interleukin-6, CRP, and ferritin were found in the HL patients, as compared to the NHL patients (7.30 (2.20–17.50) vs. 2.10 (1.20–4.20); 29.50 (1.95–54.90) vs. 2.80 (0.90–7.30); 240.5 (150.0–512.5) vs. 83.0 (28.0–240.0), respectively).

**Table 2** shown that there was no difference the values of redox, lipid and inflammatory parameters between indolent (low- grade), n=27, and aggressive (high- grade), n=20, lymphoma. Aggressive lymphomas were all Diffuse large B cell lymphoma (DLBCL), Burkitt, Mantle cell lymphoma (MCL), Peripheral T-cell lymphoma (PTCL), Anaplastic large-cell lymphoma ALCL) while other lymphomas were indolent. Also, there was no difference when comparing the biochemical parameters of follicular and DLBCL (**Supplementary Table 3**).

**Table 2:** Differences between clinical data and biochemical parameters in indolent and aggressive lymphomas.

Parameters	Indolent (n=27)	Aggressive (n=20)	*p*-value
**Male sex, n (%)**	13 (48)	11 (52.4)	0.813
**Age, years**	61 ± 14.1	63 ± 10.4	0.424
**SUVmax**	7.0 (3.25-11.25)	14.50 (2.85-21.85)	0.167
Redox parameters			
**Paraoxonase 1 (U/L)**	234 (167-487)	213 (135-370)	1.000
**TAS (mmol/L)**	994 (859-1183)	950 (772-1095)	0.500
**SH-groups (mmol/L)**	0.391 (0.271-0.457)	0.366 (0.218-0.441)	0.167
**TOS (mmol/L)**	12.3 (8.4-17.3)	13.0 (8.85-20.9)	0.667
**PAB (U/L)**	133.00 (108-152)	126.00 (108-158)	0.667
**OSI (AU)**	0.011 (0.007-0.019)	0.013 (0.009-0.024)	0.500
**AOPP (μM)**	50.7 (47.3-58.9)	54.4 (44.25-63.20)	0.667
Inflammatory parameters			
**CRP (mg/L)**	2.55 (0.87-5.20)	5.00 (0.95-12.05)	0.500
**Interleukin- 6 (pg/mL)**	2.10 (1.20-4.05)	2.00 (1.20-4.40)	0.500
**Ferritin (ng/mL)**	84 (28-237)	74 (31.00-248)	0.167
Lipid parameters			
**Cholesterol (mmol/L)**	5.13 ± 1.15	4.86 ± 1.44	0.545
**HDL-C (mmol/L)**	1.25 ± 0.45	1.10 ± 0.37	0.212
**LDL-C (mmol/L)**	3.30 ± 0.86	3.21 ± 1.29	0.899
**Triglycerides (mmol/L)**	0.66 (0.52-0.84)	0.56 (0.37-1.36)	0.667
**HDL size (nm)**	9.52 ± 0.96	9.90 ± 1.03	0.186
**HDL2b (%)**	38.9 ± 14.3	41.39 ± 11.9	0.463
**HDL2a (%)**	19.7 ± 4.9	20.86 ± 3.3	0.358
**HDL3a (%)**	13.8 ± 4.4	14.20 ± 3.4	0.629
**HDL3b (%)**	9.8 ± 3.8	8.76 ± 3.2	0.253
**HDL3c (%)**	17.7 ± 11.9	14.78 ± 11.1	0.314
**HDL3 (%)**	41.4 ± 15.7	37.76 ± 13.4	0.349
Trace element and albumin			
**Magnesium (mmol/L)**	0.79 ± 0.07	0.79 ± 0.05	0.815
**Albumin (g/L)**	45.4 ± 4.49	43.53 ± 4.94	0.109

Categorical data are presented as absolute frequencies (percentages) and analyzed by the Chi-squared test. Normally distributed data are presented as means ± SD and compared by Stu-dent’s t-test for independent data, and continuous data with skewed distribution are presented as median and interquartile range and compared by the Mann–Whitney U test; p, level of significance. TAS, total antioxidant status; TOS, total oxidation status; AOPP, advanced oxidation protein products; PAB, prooxidant–antioxidant balance; SH-groups, total protein sulfhydryl groups; OSI, oxidative stress index; CRP- C-reactive protein, LDL-C, low-density lipoprotein-cholesterol; HDL-C, high-density lipoprotein-cholesterol; HDL high-density lipoprotein; SUVmax, maximum standardized uptake value.

### 3.1 The correlation of redox parameters with the proportion of HDL subclasses and inflammatory parameters in lymphoma patients before PET scan as well as healthy subjects

**Figure 2:**
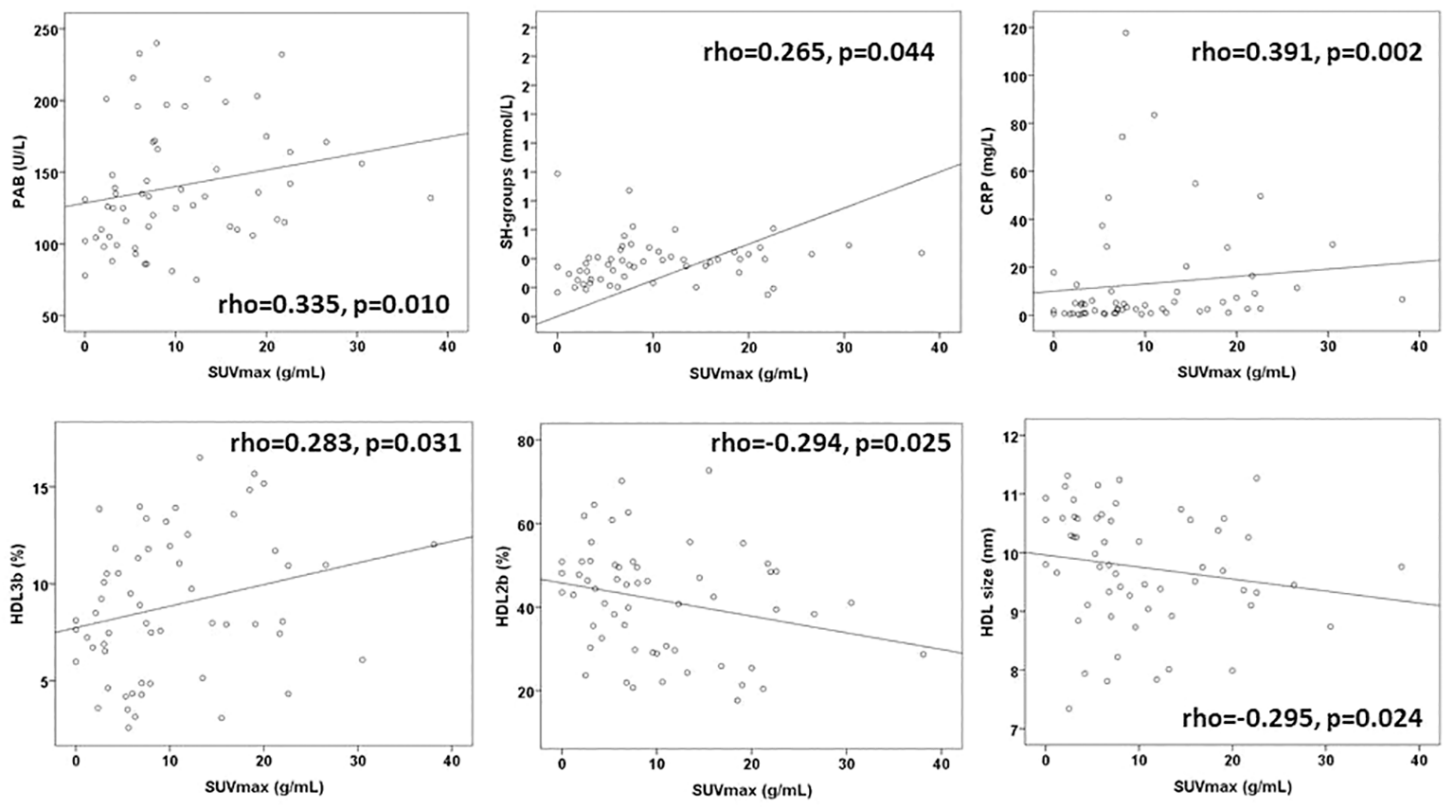
The significant correlations of biomarkers and SUVmax before therapy. PAB, prooxidant–antioxidant balance; SH-groups, total protein sulfhydryl groups; CRP, C-reactive protein, HDL, high-density lipoprotein; SUVmax, maximum standardized uptake value.

The testing correlation of the redox, lipid and the inflammatory biomarkers with SUVmax of the PET scans before therapy are shown on **Figure 2**. PAB, SH-groups, and CRP were moderately correlated with the SUVmax. Regarding the HDL subclasses, HDL3b had a positive correlation, and HDL2b and the HDL size had a negative correlation with the SUVmax.

**Supplementary Table 4** shows correlations between the observed high concentrations of redox parameters with proportions of the HDL lipoprotein subclasses and the inflammatory parameters in patients. A significantly negative correlation was observed between the SH-groups, as compared to HDL sizes and a proportion of the HDL2b subclasses, as well as in the significantly positive correlations with proportions of the HDL3b and HDL3c subclasses. Additionally, the HDL2a subclasses were positively correlated with the antioxidant paraoxonase 1and a proportion of the HDL3c subclasses, while being poorly correlated with TOS. The PAB had the strongest positive correlation with the CRP and interleukin-6. Similarly, the AOPP showed a significantly positive correlation with the CRP and interleukin-6.

Furthermore, the correlation of the antioxidants TAS, paraoxonase 1, and the SH-groups with the proportion of the HDL subclasses were investigated in 58 healthy subjects: Antioxidant TAS had a negative correlation with the HDL size and HDL2a (rho= -0.266, p=0.047; rho= -0.330, p=0.013) and was positively correlated with HDL3b, HDL3c, and the sum of the HDL3 subclasses (rho=0.300, p=0.025; rho=0.378, p=0.004, rho=0.292, p=0.029, respectively). In this group, paraoxonase1 and the SH-groups were not correlated with the proportion of the HDL subclasses.

The correlations of the inflammatory markers in patients included the following: the CRP with interleukin-6 and ferritin (rho 0.569, p<0.001; rho=0.455, p<0.001) and interleukin-6 with ferritin (rho,0.285, p=0.030).

### 3.2 The changes in redox biomarkers, HDL subclasses, and inflammatory biomarkers between two follow-up points in patients with lymphoma

**Figure 3:**
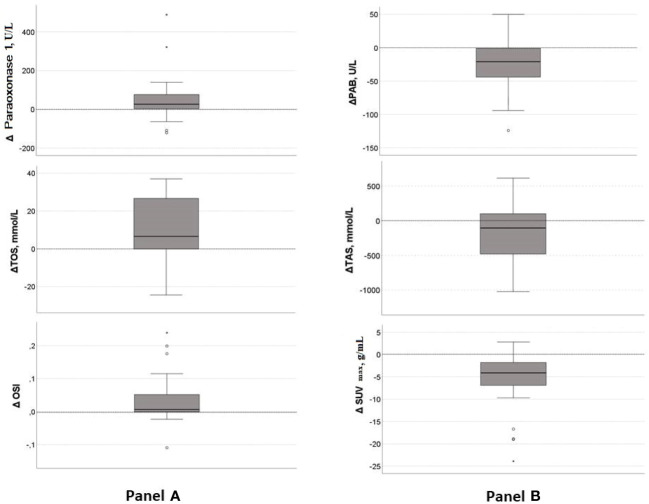
Median of difference in significantly changed parameters after chemotherapy. **(A)** –Increased parameters after treatment: TOS, OSI, paraoxonase 1; **(B)**- Reduced parameters after treatment: PAB, TAS, SUVmax. Central line denotes the median value, while the box contains values from 25th to 75th percentile. TOS, total oxidation status; OSI, oxidative stress index; PAB, prooxidant-antioxidant balance; TAS, total antioxidant status; SUVmax, maximum standardized uptake value.

The changes of redox and inflammatory parameters, along with lipid and lipoproteins, are examined in 25 patients. Paraoxonase 1, antioxidant on HDL particles, significantly increased (p=0.006), contrary to TAS, which decreased after therapy (p=0.040). Moreover, the TOS and OSI were increased (p=0.003 and p=0.016, respectively), but the PAB was reduced after treatment (p=0.012). However, the SUVmax decreased significantly after therapy, indicating a reduction in the severity of the disease (p<0.001). Medians of difference (Δ medians) for those parameters are shown in **Figure 3**. In more than 75% of patients, the paraoxonase 1 activity, OSI and TOS levels increased (Δ medians are 26 U/L, 0.066 and 6.6 mmol/L, respectively), and PAB activity decreased after therapy (Δ medians =-21U/L). However, the TAS level was reduced in less, and the SUVmax in more than 75% of patients (Δ medians are -105 mmol/L and -4.1 g/mL, respectively). None of the inflammatory or lipid parameters significantly changed after treatment.

### 3.3 Correlation of changes in examined parameters with changes of FDG-PET/CT scans

**Figure 4:**
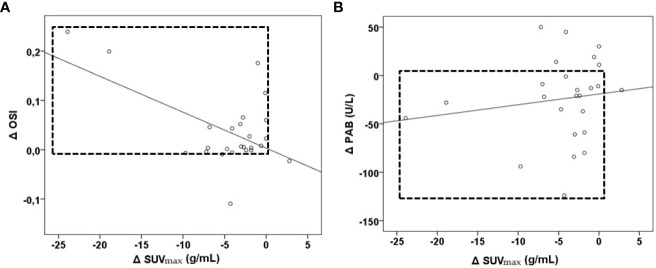
Relationship between OSI, PAB and SUVmax changes in lymphoma patients after first-line immunochemotherapy shown as part **(A)** and part **(B)**. Part **(A)**- relation between Δ SUVmax and Δ OSI, patients with reduced SUVmax after therapy had positive OSI changes. Part **(B)**- relation between Δ SUVmax and Δ PAB-patients with reduced SUVmax after therapy had negative PAB changes. Δ– the difference between values after therapy; PAB, prooxidant-antioxidant balance; OSI, oxidative stress index; SUVmax, maximum standardized uptake value.

**Figure 5:**
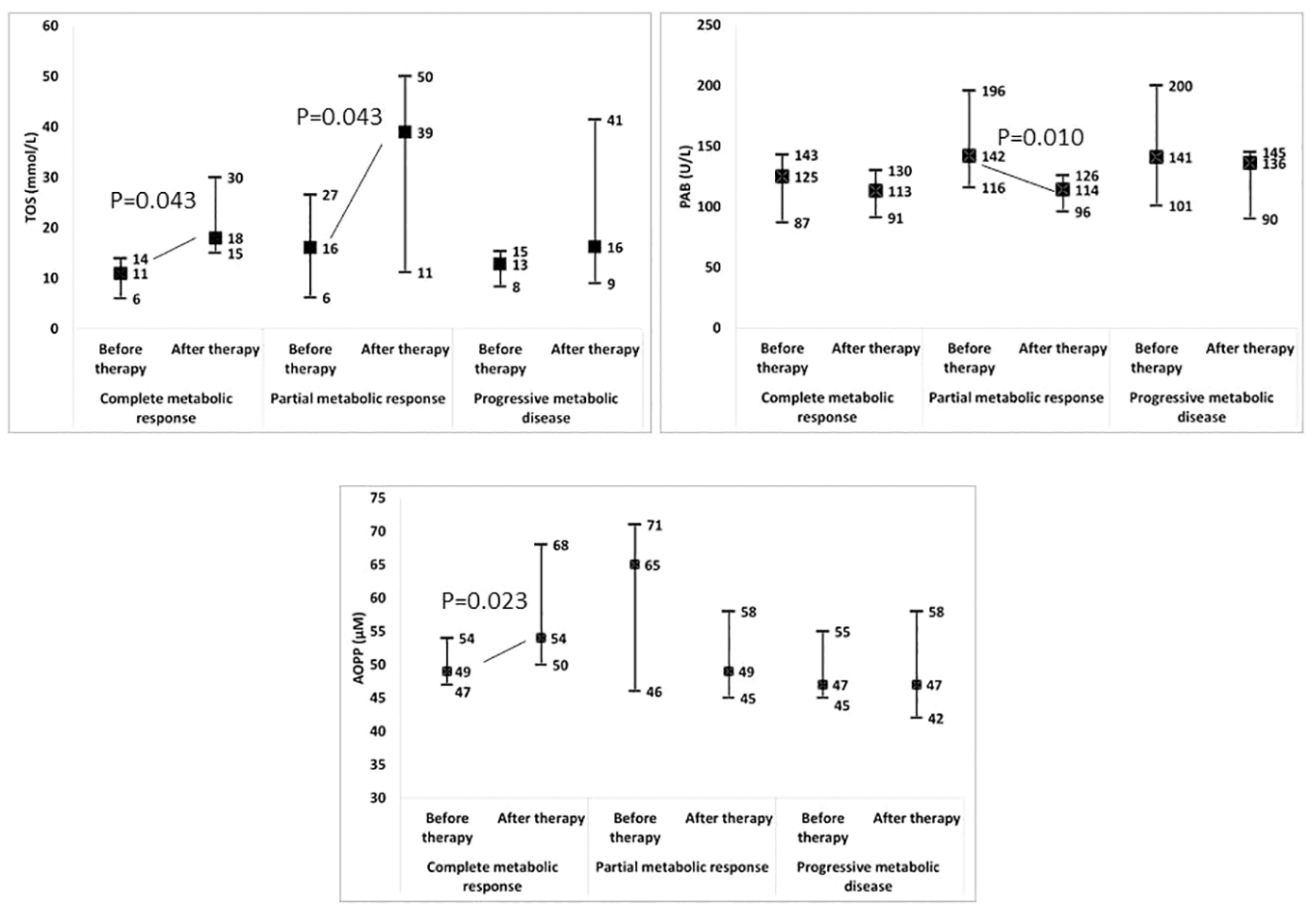
Change in redox biomarkers in relation to the response to therapy. TOS, total oxidation status; PAB, prooxidant-antioxidant balance; AOPP, advanced oxidation protein products.

Moreover, we tested whether changes in the examined parameters between the two time-points were associated with changes in the SUVmax (**Table 3**; **Supplementary Table 5**). We found that the increases in the TOS, the OSI, paraoxonase 1, the SH-groups, and the triglycerides concentrations, as well as the reductions in the TAS and the PAB, during the follow-up treatment periods were associated with the decreases in the SUVmax. Next, we added the time-varied TOS, OSI, PAB, TAS, SH-groups, and triglycerides variables into the model. The model showed that a decrease of 1 unit in the PAB and an increase of 1 unit in the OSI led to a statistically significant decrease in the SUVmax, and this was independent of any confounding conditions. **Figure 4** shows a relation between SUVmax and OSI changes. Dots in the dashed square represent OSI changes in patients with reduced SUVmax after therapy (improved SUVmax). Almost all patients with improved SUVmax after therapy had positive OSI changes i.e. OSI was increased after therapy. **Figure 4** shows a relation between SUVmax and PAB changes. Dots in the dashed square represent PAB changes in patients with reduced SUVmax after therapy (improved SUVmax). Most patients with improved SUVmax after therapy had negative PAB i.e.PAB was decreased after therapy. In additional, **Figure 5** shows the patients’ response to therapy according to the Deauville score. Significantly higher values of TOS and AOPP were observed in the group of patients who achieved a complete response to therapy, while the group of patients with a partial response had significantly lower PAB values. The 8 patients (32%) had a Deauville score of 1; 1 patient (4.00%) had a score of 2; 7 patients (28%) had a score of 3; 3 patients (12.00%) had a score of 4, and 6 patients (24.00%) had a score of 5.”

**Table 3:** Association of redox biomarkers changes wit SUVmax changes in lymphoma patients after first line immuno-chemotherapy.

Laboratory Parameters	Univariate model β (standard error)	p
Δ **TAS (mmol/L)**	0.008 (0.003)	0.018
Δ **SH-groups (mmol/L)**	-0.010 (0.004)	0.028
Δ **TOS (mmol/L)**	-0.201 (0.071)	0.010
Δ **PAB (U/L)**	0.069 (0.030)	0.032
Δ **OSI (AU)**	-60.681 (14.122)	<0.001
Δ **Triglycerides (mmol/L)**	-4.340 (1.908)	0.034
	Multivariate model β (standard error)	p
Δ **PAB (U/L)**	0.060 (0.022)	0.016
Δ **OSI**	-72.349 (29.67)	0.027

Δ - the changes between the first and second points; univariate and multivariate linear fixed-effect panel model regression was used; p, level of significance. TAS, total antioxidant status; TOS, total oxidation status; PAB, prooxidant-antioxidant balance; SH-groups, total protein sulfhydryl groups; OSI, oxidative stress index; SUVmax, maximum standardized uptake value.

## 4 Discussion

Overall, the results of our study suggested that the OS, inflammation, and lymphoma are closely related. Our study revealed significantly larger values of the TOS, the OSI, the AOPP, the PAB, the SH-groups, while showing a decrease in the TAS in the serum of patients with lymphoma, compared to healthy subjects. In addition, the reduction in the SUVmax between the two follow-up points was associated with the increase in the OSI, the TOS, and the SH-groups, as well as a reduction in the PAB and the TAS.

The OS could be present in patients with either HL or NHL as a consequence of the abnormalities in the antioxidant metabolism due to carcinogenesis. Similar results were found in colorectal, esophageal, and thyroid cancers, where the serum levels of the TOS and the OSI were higher in patients with malignant tumors while being significantly lower in healthy subjects (27–29). In addition, the TOS and the OSI were significantly higher after the completion of the first-line chemotherapy; however, the TAS was reduced, which was confirmed by previously published data on the elevated values of the redox biomarkers in cancer therapy (30). These study findings followed the results of previous studies conducted by Thanoon et al. and Abou-Seif et al. (31, 32), in which the OS had been significantly higher after chemotherapy had commenced. In addition, Zhu et al. showed that chemotherapy had reduced the antioxidant capacity in cancer patients (33).

We found a significantly higher concentration of the PAB in the sera of the patients with lymphoma and concluded that the antioxidant system had been impaired or depletion by lymphoma. Moreover, although the PAB was significantly higher in newly diagnosed patients, as compared to the healthy group, it had decreased significantly after reassessment during therapy. A small discrepancy could be observed at this point: The TOS increased (thus, the oxidative damage increased) while the PAB decreased, and both parameters reflect the concentrations of similar pro-oxidants. The TOS measured the concentration of H_2_O_2_ and lipid hydroperoxides, while PAB represented H_2_O_2_ in an antioxidant environment (*i.e*., an H_2_O_2_ surplus after its reaction with antioxidants). Since we had noticed the PAB decrease as a consequence of the H_2_O_2_ decrease (*i.e.*, its neutralization by antioxidants), the TOS increase had originated, presumably, from the lipid hydroperoxides increase. Lipid hydroperoxides are indicators of the early phase of oxidative damage in lipids, which could have been the cause. In addition, concerning the PAB, our results showed that the treatment, nevertheless, had significantly reduced this imbalance, which was visible in the positive correlation between the PAB and the changes in SUVmax. We expected that a high level of the PAB in the serum would be associated with an increased production of ROS; therefore, the results showed a strong positive correlation between the PAB and the CRP as well as interleukin-6. The observation that the antioxidant system is impaired in lymphoma patients is indeed an intriguing finding. However, it is important to consider explanations for this observation, such as depletion of antioxidant parameters by the tumor microenvironment and immune response, rather than solely attributing it to impairment. Distinguishing between these two potentials causes are crucial for developing appropriate clinical strategies. Monitoring a small number of patients at only two time points during the illness certainly limits our ability to draw reliable conclusions on this matter. Our finding could stimulate further research into the complex interplay between tumors, the immune system, and antioxidants. It may also lead to the development of novel therapies targeting these interactions.”

The AOPP was considered a reliable marker for detecting protein damage because it has been defined as a cross-linked protein product containing dityrosine (34). The AOPP values were significantly higher in HL and NHL patients, as compared to the control group. Increased values of AOPP have been found in various types of cancer, including colorectal cancer, breast cancer, and other cancers (35–37). In addition, there was a significant correlation between the AOPP and the CRP as well as interleukin-6, which was in agreement with previous studies (38, 39). In addition, the AOPP could increase the ROS formation and stimulate cytokine secretion, thereby accelerating endothelial cell injury (40). The AOPP are formed by the reaction between chlorinated oxidants (HOCl/OCl-) and proteins, and they contribute to the pathogenesis of chronic diseases and also in the regulation of inflammatory responses (41, 42).

Under stress conditions, thiol compounds are able to scavenge different toxic molecules, such as electrophilic carcinogens, which are conjugated in order to prevent tumor initiation (43). The significantly higher SH-group values in lymphoma patients, as compared to the control group was likely due to the compensatory accumulation of reduced glutathione in the tumor in response to the OS. A significant positive correlation between the SH-groups and the HDL3c subclasses at the first follow-up point in lymphoma patients, which was not found in the control group, could indicate an increased antioxidant defense in response to the cancer. In addition, the increases in the SH-groups led to a decrease in the metabolic activity of the glucose in the cells during FDG-PET scanning *(i.e*., a reduction in the SUVmax). Although no statistically significant difference was shown for SH-groups before and after therapy, that small change was related to SUVmax changes, but only in the univariate analysis. Because of that, SH-groups were included in the multivariate analysis as one of the confounders. In this case, the association with SUVmax changes was lost. The direct cause-and-effect relationships between oxidative stress parameters and changes in SUVmax are difficult to establish. In this case, the confounding factor mentioned is a response to therapy, specifically how patients respond to treatment. Future research can build upon these findings to develop a more comprehensive understanding of the relationship between OS, PET scan, and response to therapy.

The antioxidant enzyme paraoxonase 1 was also decreased in lymphoma patients, as compared to healthy people, although the difference did not reach statistical significance. Paraoxonase 1 is an enzyme involved in removing lipid peroxides generated during OS, and accumulated oxidized LDL particles inhibit the paraoxonase 1 gene expression, consequently leading to reduced paraoxonase 1 activity (44). Therefore, it was not surprising that the paraoxonase 1 activity was lower in cancer patients who had, as shown, greatly increased markers of OS. The surprisingly high value of paraoxonase 1 after the first-line immuno-chemotherapy could be explained by the protective role of paraoxonase 1 in the liver, considering that paraoxonase 1 is synthesized in the liver, where chemotherapy is also metabolized (45). The research by Abraham and Sugumar showed that increasing the level of paraoxonase 1 in the liver could be a defense mechanism to prevent or minimize CYP-induced liver damage (45).

The values of cholesterol, LDL-C, and HDL-C were significantly lower in the group of patients with lymphoma, as compared to the healthy subjects. Many research studies have shown that cholesterol synthesis is upregulated in several malignant diseases, including leukemia and lymphoma (46, 47). It was anticipated that the progression of the premalignant lesions would lead to a significant decrease in the circulating cholesterol levels. A possible cause for such a reduction in plasma cholesterol could be its increased cellular uptake, which is necessary for the progress and the proliferation of premalignant and malignant tissues (48, 49).

Currently, new therapies are being proposed that challenge the existing cancer treatment paradigms. One of the latest research approaches was presented in a study by Zhan et al., where synthetic HDL nanoparticles were used for therapeutic purposes to diffuse large B-cell lymphoma. In addition, a study by Yano et al. showed that SR-BI inhibitors were potential new anti-lymphoma therapeutics that could target cholesterol metabolism (50). This research indicated that cell survival in cancer could be halted via HDL particles, which further justified the interest in HDL subclasses. HDL particles play a pivotal role in delivering essential cholesterol for cancer cell survival, thereby promoting cancer cell growth. Conversely, they also have a protective function due to their antioxidant properties, including the presence of paraoxonase 1. However, under altered conditions such as in cancer, they can shift from their antioxidant role to exhibit prooxidant properties. Results obtained from our experimental group demonstrated that the proportions of the HDL3a and HDL3b subclasses were significantly lower, as compared to the healthy control group. The decreased proportion of the small dense particles of HDL3b and HDL3a tended to reduce the antioxidant potential of the HDL particles in lymphoma. The small, dense HDL3 particles may be superior to large, light HDL2, in terms of their capacity to prevent LDL oxidation and, thus, produce free radicals (5, 51, 52). It was interesting that in a group of healthy subjects, we found a significantly positive correlation between the TAS and the HDL3b, HDL 3c, and HDL3 particles and a significantly negative correlation with the sizes of the HDL and HDL2a particles, thus confirming that HDL3 particles had the highest anti-oxidant capacity of all the HDL particles. Our observations agreed with the research of Kontush et al. (8). The lost correlation between the TAS and the small, dense HDL3 subclasses in patients, which existed in the control group, could indicate that the HDL subclasses modified their distribution profiles under severe disease conditions. Our current observation fit with a previous conclusion that an environment with high oxidative potential could induce considerable changes in the HDL subclass distribution that could then lead to the accumulation of large HDL particles with a reduced anti-oxidative capacity.

As expected, all three examined inflammatory markers were significantly increased in lymphoma patients, as compared to healthy subjects. We have already mentioned the significant positive correlation between the AOPP and the PAB, and the markers of inflammation, and our results indicated that a reduction in the OS could also lead to a reduction in the inflammatory markers.

In our study, the levels of the serum markers interleukin -6, CRP, ferritin were significantly increased in the HL patients, as compared to the NHL patients, both before treatment, but there were no significant differences after treatment. Inflammatory cytokines, such as interleukin-6, tumor necrosis factor alpha, and so forth, are produced by the tumor micro-environment cells, which in NHL represents mostly about 10% of tumor mass, while in HL the cytokines are produced not only by large amount of tumor microenvironment cells but also by the Hodgkin and Reed–Sternberg cells (53, 54). These aspects could explain the results we obtained when comparing biochemical markers between HL and NHL patients. There were no statistically significant differences in these parameters when the patients were reassessed due to the reduced number of tumor cells after therapy (*i.e*., a reduced SUVmax), as interleukin-6, CRP, and ferritin had also been reduced.

In addition, the lower level of magnesium in the serum of our patients with lymphoma indicated that magnesium could be considered as a novel element in the pathogenesis of lymphoma. It is still not clear whether low magnesium is a consequence of lymphoma or contributes to the development of lymphoma (15, 16). Further investigation is needed to understand the role of magnesium in preventing lymphoma and improving the outcomes of patients with lymphoma and concomitant magnesium deficiency.

All together, statistically significantly lower SUVmax values at the time of reassessment indicate a reduced number of metabolically active or ‘live’ tumor cells, thus suggesting a favorable therapeutic effect of the administered chemotherapy protocols, which include cytostatic such as doxorubicin and cyclophosphamide that predominantly exhibit pro-oxidative effects and kill cancer cells through direct or indirect ROS overaccumulation. This is supported by the obtained results showing elevated OSI and TOS values, along with reduced PAB and TAS values. An increase in ROS leads to the activation of different cell death pathways in tumor cells, thus limiting cancer progression (55, 56).

Although the values of inflammatory markers CRP and interleukin-6 were lower at the point of reassessment, this difference did not reach statistical significance, which could, on the one hand, be attributed to the fact that elevated ROS activate inflammatory pathways and the release of inflammatory markers. Oxidative stress markers might show significant changes earlier due to the immediate effects of treatments, while inflammatory or lipid parameters might take longer to manifest changes. Some changes may normalize over time, and this could explain the less pronounced effects on inflammatory and lipid parameters in our study. Also, the higher ferritin values at the point of reassessment, without statistical significance, could be interpreted as a consequence of red blood cell transfusion in our patients.

The levels of paraoxonase 1 on HDL particles, TOS, and OSI increased, while TAS and PAB decreased after therapy. However, only changes in OSI and PAB were independently related to SUVmax changes during therapy, irrespective of other confounding variables. An increase in OSI after therapy was associated with a decrease in SUVmax. On the other hand, a decrease in PAB after therapy was also linked to a decrease in SUVmax. When analyzing the redox parameters with the Deauville score, it can be observed that patients who exhibited significantly increased oxidative stress (increased TOS and AOPP) and significantly lower PAB achieved a complete or partial response to therapy. Conversely, patients whose therapy did not result in increased oxidative stress experienced disease progression. These results from our study raise the question of concurrent intake of antioxidants and cancer therapy, whether antioxidants provide more benefits or harm.

The limitations of our study included having a relatively small number of participants, which was further compounded by the loss of participants at the second point of assessment due to the emergence of COVID-19 and the numerous epidemiological measures implemented in our country. Also, in the NHL group differences in tumor cells origin, their metabolism and biology probably could explain obtained results. In addition, due to the small number of samples, we could not analyze each tumor stage individually. However, with such studies, the question remains whether the observed parameters were the cause or the consequence of the disease state under investigation. Ongoing and future studies on lymphoma will likely provide more context for our results.

In conclusion, the redox parameters in patients with lymphoma were consistent with FDG-PET/CT findings. Our results showed increased oxidative stress and decreased antioxidant response in patients with lymphoma, indicating that oxidative stress plays an important role in lymphoma carcinogenesis. Patients who experienced a significant increase in oxidative stress during therapy showed a complete metabolic response to the therapy Excessive production of oxidative stress in malignant cells due to therapy could block the antioxidant shield, provided by the tumor microenvironment. In view of the fact that tumor biology still remains poorly understood, future experimental and clinical studies of the role of ROS are essential for deepening understanding of cancer pathology. Lastly, extensive research should also consider the ROS biomarkers and HDL subclasses as targets in order to pave the way for superior lymphoma therapy.

## Data availability statement

The original contributions presented in the study are included in the article/**Supplementary Material**. Further inquiries can be directed to the corresponding author.

## Ethics statement

The studies involving humans were approved by Ethics Committee of University Clinical Centre of the Republic of Srpska, Banja Luka (No 01-19-51-2/20) and at the Faculty of Medicine at the University of Banja Luka (No 18/4.3.95/2020). The studies were conducted in accordance with the local legislation and institutional requirements. The participants provided their written informed consent to participate in this study.

## Author contributions

BM-A, NB-S, ZRS and SS designed the study. NB-S performed the statistical analysis. BM-A, DM-Z and SS recruited and screened the participants. DM-Z and SS diagnosed the patients. BM-A, ZRS, DM-Z, LN performed the literature search and wrote the manuscript. RS and JK-S gave critical comments to the manuscript. All authors contributed to the article and approved the submitted version.
