# Supplementary material for: Analysis of redox status and HDL subclasses in patients with lymphoma and the associations with FDG-PET/CT findings
Source: Front Oncol. 2023 Oct 30;13:1221414. doi: 10.3389/fonc.2023.1221414 (PMC10642952; doi:10.3389/fonc.2023.1221414)
Supplement: Supplementary file 1 [file DataSheet_1.pdf]

Supplementary Material

Analysis of redox status and HDL subclasses in patients with lymphoma and the associations with FDG-PET/CT findings

Bosa Mirjanic-Azaric[1,2*], Sinisa Stankovic[3,4], Lana Nezic[5], Zana Radic Savic[1], Dragana Malcic-Zanic[6], Ranko Skrbic[5,7], Jelena Kotur-Stevuljevic[8] and Natasa Bogavac-Stanojevic[8]

[1] Department of Medical Biochemistry, Faculty of Medicine, University of Banja Luka, Banja Luka, The Republic of Srpska, Bosnia and Herzegovina

[2] Institute of Laboratory Diagnostic, University Clinical Centre of the Republic of Srpska, Banja Luka, The Republic of Srpska, Bosnia and Herzegovina

[3] Institute of Nuclear Medicine, University Clinical Centre of the Republic of Srpska, Banja Luka, The Republic of Srpska, Bosnia and Herzegovina

[4] Faculty of Medicine, University of Banja Luka, Banja Luka, Republic of Srpska, Bosnia and Herzegovina;

[5] Department of Pharmacology, Toxicology and Clinical Pharmacology, Faculty of Medicine, University of Banja Luka, Banja Luka, The Republic of Srpska, Bosnia and Herzegovina

[6] Department of Pediatrics, Faculty of Medicine, University of Banja Luka, Banja Luka, The Republic of Srpska, Bosnia and Herzegovina

[7] Academy of Sciences and Arts of the Republic of Srpska, Banja Luka, The Republic of Srpska, Bosnia and Herzegovina

[8] Department of Medical Biochemistry, University of Belgrade-Faculty of Pharmacy, Belgrade, Serbia

*** Correspondence:**
Bosa Mirjanic-Azaric
bosa.mirjanic@med.unibl.org

1 Supplementary Tables

Supplementary Table 1. Normally distributed laboratory parameters of study population prior to treatment

Parameters	Patients (n=58)	Control group (n=58)	p-value
**Lipid parameters**			

**Cholesterol (mmol/L)**	4.78 ± 0.149	5.55 ± 0.149	0.001
**HDL-C (mmol/L)**	1.16 ± 0.053	1.39 ± 0.53	0.003
**LDL-C (mmol/L)**	3.07 ± 0.137	3.59 ±0.137	0.010
**HDL size (nm)**	9.74 ± 0.126	9.54 ± 0.129	0.300
**HDL2b (%)**	42.7 ± 1.583	37.6 ± 1.613	0.058
**HDL2a (%)**	20.6 ± 0.735	21.6 ± 0.749	0373
**HDL3a (%)**	13.7 ± 0.622	17.1 ± 0.634	<0.001
**HDL3b (%)**	8.8 ± 0.511	10.6 ± 0.521	0.013
**HDL3c (%)**	14.8 ± 1.232	12.8 ± 1.255	0.267
**HDL3 (%)**	37.5 ± 1.801	40.6 ± 1.834	0.205
**Trace element and albumin**			
**Magnesium (mmol/L)**	0.79 ± 0.009	0.83 ± 0.013	0.027
**Albumin (g/L)**	44.4 ± 4.84	47.0 ± 2.78	0.002

Data are presented as adjusted means and standard error and compared by ANCOVA analyses. Means are adjusted for age.

Supplementary Table 2. F and p values from Quade's test output for comparison of redox and inflammatory parameters and triglycerides between healthy and lymphoma subjects.

**Parameters**	F	p
**Paraoxonase 1 (U/L)**	3.094	0.081
**TAS (mmol/L)**	13.147	<0.001
**SH-groups (mmol/L)**	14.710	<0.001
**TOS (mmol/L)**	8.606	0.004
**PAB (U/L)**	69.940	<0.001
**OSI (AU)**	10.622	<0.001
**AOPP (µM)**	15.743	<0.001
**CRP (mg/L)**	30.915	<0.001
**Interleukin-6 (pg/mL)**	16.045	<0.001
**Ferritin (ng/mL)**	20.312	<0.001
**Triglycerides (mmol/L)**	19.678	<0.001

Abbreviations: TAS, total antioxidant status; SH-groups, total protein sulfhydryl groups; TOS, total oxidation status; PAB, prooxidant-antioxidant balance; OSI, oxidative stress index; AOPP, advanced oxidation protein products; CRP, C-reactive protein.

Supplementary Table 3. Differences between clinical data and biochemical parameters in follicular lymphoma and DLBCL patients

Parameters	Follicular lymphoma (n=21)	DLBCL (n=14)	p-value
**Male sex, n (%)**	10 (47.6)	8 (57.1)	0.581
**Age, years**	59 ± 14.6	63 ± 10.1	0.334
**SUVmax**	7. (3.75-12.10)	17.55 (4.87-22.60)	0.061
**Redox parameters**			
**Paraoxonase 1 (U/L)**	234 (167-487)	288 (183-538)	0.706
**TAS (mmol/L)**	1023 (845-1196)	941 (700-1152)	0.396
**SH-groups (mmol/L)**	0.394 (0.298-0.467)	0.366 (0.218-0.441)	0.434
**TOS (mmol/L)**	13.5 (9.35-18.0)	13.85 (6.30-23.82)	0.803
**PAB (U/L)**	135.00 (115-169)	124.00 (103-144)	0.235
**OSI (AU)**	0.013 (0.007-0.019)	0.014 (0.006-0.032)	0.702
**AOPP (µM)**	51.2 (48.1-54.9)	55.9 (44.10-64.97)	0.538
**Inflammatory parameters**			
**CRP (mg/L)**	2.60 (1.00-5.85)	2.75 (0.80-13.00)	0.855
**Interleukin- 6 (pg/mL)**	2.00 (1.15-4.10)	1.95 (1.07-3.67)	0.855
**Ferritin (ng/mL)**	85 (25-270)	110 (38-264)	0.829
**Lipid parameters**			
**Cholesterol (mmol/L)**	5.15 ± 1.15	5.14 ± 1.49	0.978
**HDL-C (mmol/L)**	1.18 ± 0.36	1.14 ± 0.37	0.701
**LDL-C (mmol/L)**	3.38 ± 0.80	3.42 ±1.25	0.920
**Triglycerides (mmol/L)**	0.65 (0.49-0.81)	0.59 (0.42-1.00)	0.630
**HDL size (nm)**	9.39 ± 0.92	9.93 ± 0.91	0.104
**HDL2b (%)**	34.9 ± 12.5	41.6 ±9.2	0.096
**HDL2a (%)**	19.5 ± 5.4	21.9 ± 2.2	0.124
**HDL3a (%)**	14.2 ± 4.5	15.1 ± 3.6	0.543
**HDL3b (%)**	10.7 ± 3.7	8.6 ± 2.6	0.080

**HDL3c (%)**	20.6 ± 11.5	12.7 ±6.2	0.025
**HDL3 (%)**	45.5 ± 14.3	36.4 ± 8.3	0.040
**Trace element and albumin**			
**Magnesium (mmol/L)**	0.79 ± 0.07	0.79 ± 0.04	0.909
**Albumin (g/L)**	44.5 ± 4.39	41.53 ± 6.94	0.089

Categorical data are presented as absolute frequencies (percentages) and analyzed by the Chi-squared test. Normally distributed data are presented as means ± SD and compared by Student's t-test for independent data, and continuous data with skewed distribution are presented as median and interquartile range and compared by the Mann–Whitney U test; p, level of significance; TAS, total antioxidant status; TOS, total oxidation status; AOPP, advanced oxidation protein products; PAB, prooxidant–antioxidant balance; SH-groups, total protein sulfhydryl groups; OSI, oxidative stress index; CRP, C-reactive protein, LDL-C, low-density lipoprotein-cholesterol; HDL-C, high-density lipoprotein-cholesterol; HDL high-density lipoprotein; SUVmax, maximum standardized uptake value.

Supplementary Table 4. The correlation of redox biomarkers with a proportion of the HDL subclasses and inflammatory parameters in 58 patients with lymphoma before therapy.

Laboratory parameters	HDL-C mmol/L Rho (p)	HDL size (nm) Rho (p)	HDL2b (%) Rho (p)	HDL2a (%) Rho (p)	HDL3a (%) Rho (p)	HDL3b (%) Rho (p)	HDL3c (%) Rho (p)	HDL3 (%) Rho (p)	CRP (mg/L) Rho (p)	IL-6 (pg/mL) Rho (p)	Ferritin (ng/mL) Rho (p)
**Paraoxonase 1 (U/L)**	0.098 (0.491)	0.113 (0.424)	0.082 (0.565)	0.305, (0.028)	0.269 (0.054)	-0.029 (0.837)	-0.157 (0.267)	-0.142 (0.314)	-0.050 (0.723)	0.040 (0.777)	-0.162 (0.250)
**TAS (mmol/L)**	-0.049 (0.712)	-0.083 (0.535)	-0.050 (0.710)	-0.040 (0.767)	0.084 (0.531)	0.128 (0.340)	-0.009 (0.946)	0.076 (0.570)	0.021 (0.873)	-0.134 (0.314)	0.016 (0.906)
**SH-groups (mmol/L)**	0.081 (0.543)	-0.379 (0.003)	-0.317 (0.015)	0.040 (0.764)	-0.122 (0.361)	0.277 (0.036)	0,349 (0.007)	0.220 (0.097)	0.053 (0.692)	-0.035 (0.796)	0.098 (0.466)
**TOS (mmol/L)**	-0.233 (0.078)	-0.140 (0.296)	-0.226 (0.088)	-0.031 (0.817)	0.033 (0.806)	0.156 (0.241)	0.260 (0.048)	0.190 (0.154)	0.107 (0.425)	0.034 (0.802)	0.158 (0.237)
**PAB (U/L)**	-0.289 (0.028)	0.110 (0.413)	0.124 (0.352)	0.007 (0.961)	-0.101 (0.450)	-0.102 (0.445)	-0.190 (0.153)	-0.188 (0.158)	0.726 (<0.001)	0.386 (0.003)	0.150 (0.260)
**OSI (AU)**	-0.148 (0.266)	-0.038 (0.776)	-0.112 (0.401)	-0.047 (0.729)	-0.051 (0.706)	0.049 (0.713)	0.177 (0.183)	0.084 (0.529)	0.087 (0.515)	0.092 (0.491)	0.120 (0.370)
**AOPP (µM)**	-0.272 (0.039)	0.005 (0.970)	-0.057 (0.671)	-0.029 (0.829)	0.012 (0.928)	0.004 (0.975)	0.035 (0.792)	0.010 (0.941)	0.395 (0.002)	0.292 (0.026)	0.245 (0.064)

Rho, Spearman’s correlation coefficient; p, level of significance. TAS, total antioxidant status; TOS, total oxidation status; AOPP, advanced oxidation protein products; PAB, prooxidant–antioxidant balance; SH-groups, total protein sulfhydryl groups; OSI, oxidative stress index; CRP, C-reactive protein; HDL-C, high-density lipoprotein-cholesterol; HDL high-density lipoprotein.

Supplementary Table 5. Association between redox biomarkers, HDL subclasses, and inflammatory biomarker changes, and SUVmax changes in lymphoma patients after first-line immuno-chemotherapy.

Laboratory Parameters	Univariate model β (standard error)	*p*-value
**Δ Paraoxonse 1 (U/L)**	-0.013 (0.012)	0.274
**Δ TAS (mmol/L)**	0.008 (0.003)	0.018
**Δ SH-groups (mmol/L)**	-0.010 (0.004)	0.028
**Δ TOS (mmol/L)**	-0.201 (0.071)	0.010
**Δ PAB (U/L)**	0.069 (0.030)	0.032
**Δ OSI (AU)**	-60.681 (14.122)	<0.001
**Δ AOPP (μM)**	0.068 (0.121)	0.577
**Δ CRP (mg/L)**	0.002 (0.042)	0.966
**Δ Interleukin-6 (pg/mL)**	-0.051 (0.136)	0.712
**Δ Ferritin (ng/mL)**	-0.003 (0.003)	0.466
**Δ Cholesterol (mmol/L)**	0.287 (1.807)	0.875
**Δ HDL-C (mmol/L)**	-2.193 (5.770)	0.708
**Δ LDL-C (mmol/L)**	0.749 (2.273)	0.745
**Δ Triglycerides (mmol/L)**	-4.340 (1.908)	0.034
**Δ HDL size (nm)**	0.167 (1.645)	0.920
**Δ HDL2b (%)**	0.074 (0.394)	0.853
**Δ HDL2a (%)**	0.813 (0.766)	0.301
**Δ HDL3a (%)**	-1.282 (0.680)	0.073
**Δ HDL3b (%)**	-0.828 (0.499)	0.112
**Δ HDL3c (%)**	0.588 (0.395)	0.152
**Δ HDL 3 (%)**	-0.285 (0.382)	0.463
**Δ Magnesium (mmol/L)**	-3.595 (17.103)	0.835
**Δ Albumin (g/L)**	0.281 (0.375)	0.468
	**Multivariate model β (standard error)**	***p*-value**
**Δ TAS (mmol/L)**	-0.002 (0.010)	0.876
**Δ SH-groups (mmol/L)**	-0.003 (0.005)	0.508
**Δ TOS (mmol/L)**	0.114 (0.188)	0.552
**Δ PAB (U/L)**	0.060 (0.022)	0.016
**Δ OSI (AU)**	-72.349 (29.67)	0.027
**Δ Triglycerides (mmol/L)**	-1.895 (1.574)	0.508

Δ - the changes between the first and second points; univariate and multivariate linear fixed-effect panel model regression was used; p, level of significance. TAS, total antioxidant status; TOS, total oxidation status; AOPP, advanced oxidation protein products; PAB, prooxidant-antioxidant balance; SH-groups, total protein sulfhydryl groups; OSI, oxidative stress index; AU, arbitrary unit; CRP, C-reactive protein, LDL-C, low-density lipoprotein-cholesterol; HDL-C, high-density lipoprotein-cholesterol; HDL high-density lipoprotein; SUVmax, maximum standardized uptake value.
